# Pf Filamentous Phage Requires UvrD for Replication in *Pseudomonas aeruginosa*

**DOI:** 10.1128/mSphere.00104-15

**Published:** 2016-02-10

**Authors:** Eriel Martínez, Javier Campos-Gómez

**Affiliations:** Department of Infectious Diseases, Drug Discovery Division, Southern Research, Birmingham, Alabama, USA; University of Michigan

**Keywords:** *Pseudomonas aeruginosa*, Pf filamentous phage, UvrD helicase, Rep helicase, histone-like HU, rolling circle replication

## Abstract

Pf is a lysogenic filamentous phage that promotes biofilm development in *Pseudomonas aeruginosa*. Pf replicates by a rolling circle replication system which depends on a phage-encoded initiator protein and host factors usually involved in chromosome replication. Rep, an accessory replicative DNA helicase, is crucial for replication of filamentous phages in *Escherichia coli*. In contrast, here we show that, instead of depending on Rep, Pf replication depends on UvrD, an accessory helicase implicated in DNA repair. In this study, we also identified the initiator protein of Pf and found that it shares similarities with that of *Vibrio* phages CTXφ and VGJφ, which also depend on UvrD for replication. A structural comparative analysis of the initiator proteins of most known filamentous phages described thus far suggested that UvrD, known as a nonreplicative helicase, is involved in rolling circle replication of filamentous phages in diverse bacteria genera. This report consolidates knowledge on the new role of UvrD in filamentous phage replication, a function previously thought to be exclusive of Rep helicase.

**IMPORTANCE** Biofilm development is a key component of the ability of *Pseudomonas aeruginosa* to evade host immune defenses and resist multiple drugs. Induction of the filamentous phage Pf, which usually is lysogenized in clinical and environmental isolates of *P. aeruginosa*, plays an important role in biofilm assembly, maturation, and dispersal. Despite the clinical relevance of Pf, the molecular biology of this phage is largely unknown. In this study, we found that rolling circle replication of Pf depends on UvrD, a DNA helicase normally involved in DNA repair. We also identified the initiator protein of Pf and found that it shares structural similarity with that of *Vibrio cholerae* phages CTXφ and VGJφ, which also use UvrD for replication. Our results reveal that, in addition to DNA repair, UvrD plays an essential role in rolling circle replication of filamentous phages among diverse bacteria genera, adding a new, previously unrecognized function of this accessory helicase.

## Abstract (precis)

Biofilm development is a key component of the ability of *Pseudomonas aeruginosa* to evade host immune defenses and resist multiple drugs. Induction of the filamentous phage Pf, which usually is lysogenized in clinical and environmental isolates of *P. aeruginosa*, plays an important role in biofilm assembly, maturation, and dispersal. Despite the clinical relevance of Pf, the molecular biology of this phage is largely unknown. In this study, we found that rolling circle replication of Pf depends on UvrD, a DNA helicase normally involved in DNA repair. We also identified the initiator protein of Pf and found that it shares structural similarity with that of *Vibrio cholerae* phages CTXφ and VGJφ, which also use UvrD for replication. Our results reveal that, in addition to DNA repair, UvrD plays an essential role in rolling circle replication of filamentous phages among diverse bacteria genera, adding a new, previously unrecognized function of this accessory helicase.

## INTRODUCTION

*Pseudomonas aeruginosa* is a highly versatile opportunistic pathogen which is the leading cause of morbidity and mortality among cystic fibrosis (CF) patients and causes significant infection in other immunocompromised humans (1). The capacity of *P. aeruginosa* to form recalcitrant biofilms is one of the main causes of the establishment and persistence of this bacterium in chronic infection of CF patients (2). The biofilm increases the bacterium’s ability to evade host immune defenses and helps to protect the bacteria against exogenous antibiotic treatment (3). Increasing evidence suggests that the members of the family of Pf1-like filamentous bacteriophages (Pf), widely distributed among clinical and environmental strains of *P. aeruginosa*, decisively contribute to biofilm development (4, 5).

Filamentous phages belong to the *Inovirus* genus of the *Inoviridae* family of phages, which are long and slender proteinaceous tubes encasing a positive-sense single-stranded circular DNA (6). Pf is a member of the inoviruses that is usually integrated into the chromosome of *P. aeruginosa* (7), but it also can replicate without integrating, as is the case with Pf1 variant in the PAK host strain (8). The integration mechanism used by Pf phage for lysogenization is unknown, but it seems to be different from those of the filamentous phages thus far described. While other filamentous phages hijack XerCD recombinases of their bacterial hosts to integrate into the *dif* site of the bacterial chromosome (9), Pf phage encodes its own integrase, which it probably uses for lysogenization.

Pf phage-carried genes are strongly upregulated in biofilm cells (10). The prophage is also induced during development of biofilms, which release phage particles into the extracellular media (5). Recently, it was described that extracellular Pf particles promote biofilm assembly and function by interacting with infected host and bacterial biopolymers to form higher-order crystal structures that enhance biofilm adhesion, desiccation survival, and antibiotic resistance (4). Interestingly, a variant of Pf formed in mature biofilms is able to kill the bacterial host, a characteristic rarely observed among filamentous phages (5). It has been reported that the extracellular DNA released by Pf-mediated bacterial lysis functions as a structural support for the biofilm architecture. In addition, Pf-mediated cell death is an important mechanism of differentiation inside microcolonies that facilitates dispersal of subpopulations of surviving cells (11). It is also interesting that the Pf variant produced in biofilm is able to infect *P. aeruginosa* even when the strain contains an integrated copy of the same phage, circumventing the phage immunity normally provided by the inserted copy of the Pf lysogen. Therefore, this variant of Pf has been named superinfective phage (SI Pf) (5).

Pf replicates using a rolling circle replication (RCR) mechanism, which produces the single-stranded DNA genome of the phage (6). Replication starts with the binding of the phage-encoded initiator protein (IP) at a specific site in the replicative form of the phage, the *ori*(*+*) (6). The IP introduces a nick, creating a 5′-phosphotyrosine intermediate and a free 3′-OH at the *ori*(*+*). The rest of the process is driven by host factors that are usually essential for replication of the bacterial chromosome together with the IP forming the phage replisome. The replisome starts polymerizing at the free 3′-OH, and when it makes a complete round, the IP circularizes the original positive DNA strand that was displaced by the *de novo* synthesized DNA, and a new replication cycle then follows. Instead of DnaB, the bacterial helicase essential for chromosomal replication, filamentous phage replication depends on the replicative Rep helicase in *Escherichia coli* (12). Rep is an accessory helicase normally involved in chromosomal replication that directly interacts with DnaB and helps advance the replisome by removing nucleoprotein complexes in front of the replication fork (13, 14). In contrast, we recently reported that replication of *Vibrio cholerae* filamentous phages CTXφ and VGJφ depends on another accessory helicase, the DNA repair UvrD helicase (12). UvrD is well known for its role in UvrABC-dependent nucleotide excision repair (15) and MutHLS-dependent mismatch DNA repair (16). The role of UvrD in bacterial chromosome replication seems to be more limited than the role of Rep. UvrD can promote the movement of the replisome along protein-bound DNA and participates in the restart of replication forks (14), but deletion of the uvrD gene does not directly affect replication fork progression in *E. coli* (13).

Here we report for the first time that replication of Pf phage depends on UvrD helicase. UvrD is encoded by the open reading frame (ORF) PA5443 (a homologue of *E. coli* UvrD) in *P. aeruginosa*, and we show here that it is a fully functional homologue of UvrD of *E. coli*. We also found that, as with the CTXφ and VGJφ phages, histone-like protein HU is a host factor implicated in Pf replication. Additionally, we demonstrated that PA0727, an ORF localized upstream of the putative phage integrase, encodes the initiator protein of Pf. Although the amino acid sequence of the IP of Pf has no homology with that of any other described IP, an analysis revealed that it shares structural similarities with the IPs of *Vibrio* phages relying on UvrD for replication and with the IPs of many filamentous phages of other bacterial species. These findings consolidate information concerning the new function for UvrD helicase in filamentous phage replication, making UvrD a more versatile helicase than previously thought.

## RESULTS

### Pf phage is spontaneously produced in a MutS-deficient genetic background.

**FIG 1:**
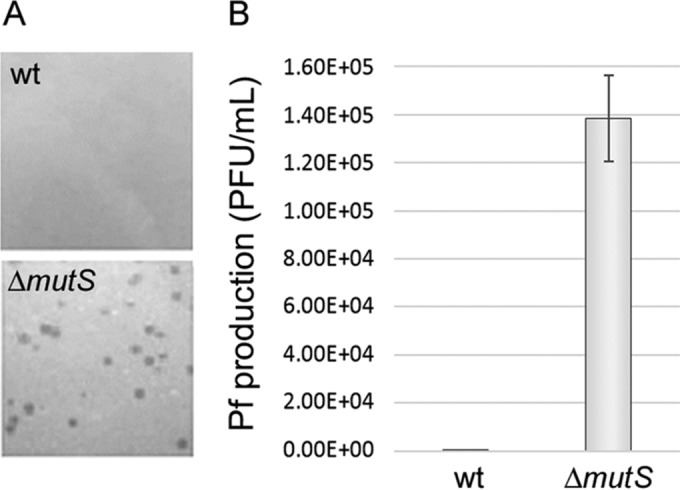
The *P. aeruginosa* Δ*mutS* mutant spontaneously produces superinfective Pf particles. (A) The wild-type PAO1 strain (wt) and the Δ*mutS* mutant were plated to confluency on LB agar plates; only the Δ*mutS* mutant produced clearly visible phage plaques spontaneously on the bacterial lawn. (B) Overnight cultures of the Δ*mutS* mutant spontaneously produced more than 10^5^ SI-Pf particles/ml, while the wt strain did not produce detectable amounts of Pf. The PAO1 strain was used for titration of the phage. Data represent means and standard deviations of the results of 3 independent experiments.

In the PAO1 model strain, a superinfective variant of Pf (SI Pf) is normally exported to the extracellular media during the dispersal phase of biofilms (5). Previous studies have reported some physiological and genetic host factors involved in the formation of SI Pf (17). It was reported that a mismatch repair (MMR)-deficient strain with a *mutS* deletion resulted in the early appearance and increased numbers of SI-Pf in biofilms of *P. aeruginosa* (17). Here, we show that a deletion of the *mutS* gene (Table 1) induces spontaneous production of SI Pf even under planktonic conditions of growth. SI Pf can be detected because it forms plaques on lawns of *P. aeruginosa*. Numerous plaques generated by SI Pf were spontaneously formed in lawns of the *ΔmutS* mutant but not in strain PAO1 (Fig. 1A). The quantity of SI Pf produced by the *ΔmutS* mutant was on the order of 10^5^ particles/ml in an overnight culture (with titers determined using PAO1 as host strain), while no SI-PF particles were detected in the supernatant of the wt PAO1 parental strain grown under the same conditions (Fig. 1B).

**TABLE 1:** Bacterial strains used in this study

Strain	Genotype/phenotype^a^	Reference or source
*P. aeruginosa*		
PAO1	Wild type	6
EMP1	PAO1, Δ*mutS*, deletion from aa 188 to 501 of 806 aa	This study
EMP2	PAO1, Δ*uvrD*, deletion from aa 16 to 717 of 731 aa	This study
EMP3	PAO1, Δ*rep*, deletion from aa 10 to 646 of 669 aa	This study
EMP4	PAO1, Δ*mutS* Δ*uvrD* (same as above for each gene)	This study
EMP5	PAO1, ΔPA0727, deletion from aa 16 to 392 of 431 aa	This study
EMP6	PAO1, Δ*mutS* ΔPA0727 (same as above for each gene)	This study
EMP7	PAO1, PA2077 Y → F	This study
EMP8	PAO1, Δ*mutS*, PA0727 Y → F	This study
PW10010	PAO1 *hupA*::ISlacZ/hah	20
PW4171	PAO1 *hupB*::ISphoA/hah	20
*E. coli*		
DH5α	*fhuA2 lac*Δ *U169 phoA glnV44* Φ*80*′ *lacZ*Δ M15 *gyrA96 recA1 relA1 endA1 thi-1 hsdR17*	Invitrogen
S17.1 λpir	Tp^r^ Sm^r^ *recA thi pro hsdR*−M+RP4::2-Tc::Mu::*km* Tn*7* λpir	35

a aa, amino acid; Sm^r^, streptomycin resistance; Tp^r^, trimethoprim resistance.

### UvrD promotes UvrABC-dependent nucleotide excision repair in *P. aeruginosa*.

**FIG 2:**
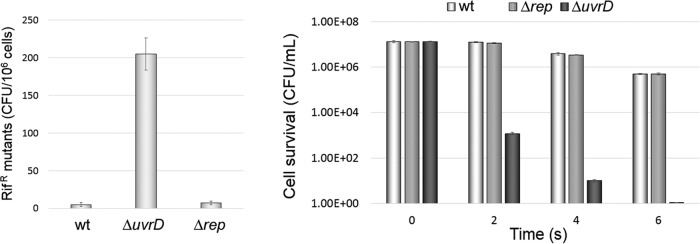
UvrD protein mediates mismatch repair and UvrABC-dependent nucleotide excision repair in *P. aeruginosa*. (A) The spontaneous appearance of rifampin-resistant cells was more than 29-fold and 40-fold higher for the Δ*uvrD* mutant than for the Δ*rep* mutant and the wt PAO1 strain, respectively. (B) The susceptibility of wt PAO1 and its isogenic Δ*rep* and Δ*uvrD* mutants to the UV radiation was tested by directly exposing suspensions of cells to a UV light source for 0, 2, 4, and 6 s. At 2 s of UV exposure, more than 80% of wt and Δ*rep* mutant cells were able to survive, while less than 0.01% of the Δ*uvrD* mutant cells could survive the irradiation. Data represent means and standard deviations of the results of 3 independent experiments.

The genome of the PAO1 strain encodes a homologue of *E. coli* UvrD (PA5443); however, UvrD has not been fully characterized in *P. aeruginosa*. It was previously reported that a *P. aeruginosa* isolate with a mutated PA5443 gene, which produces a variant of UvrD with 3 amino acid substitutions within the conserved ATP binding site, showed defective MMR activity (18). Unexpectedly, the strain showed the same UV sensitivity as the wt strain, casting doubts on the role of the PA5443 product in UvrABC-dependent nucleotide excision repair (NER) (18). To ascertain that the product of PA5443 encodes a functional homologue of *E. coli* UvrD helicase, we deleted the PA5443 gene from the PAO1 strain (Table 1). Consistent with a role of PA5443 product in MMR, the ΔPA5443 mutant showed a hypermutagenic phenotype. This mutant generated approximately 40-fold and 29-fold more spontaneous rifampin-resistant (Rif^r^) colonies than a *rep* deletion mutant (Table 1) and the wild-type strain, respectively (Fig. 2A). Subsequently, the role of the PA5443 product in NER was revealed by the increased sensitivity of the ΔPA5443 mutant to UV radiation. The ΔPA5443 mutant was 4 orders of magnitude more sensitive to UV radiation than a Rep mutant and the wt PAO1 strain (Fig. 2B). A Rep mutant was included as a control, since it is a helicase of the same family (SF1) as UvrD, which is normally involved in replication but not in DNA repair. Our results indicate that the PA5443 gene encodes a *P. aeruginosa* functional homologue of *E. coli* UvrD helicase.

### Pf replication depends on UvrD.

**FIG 3:**
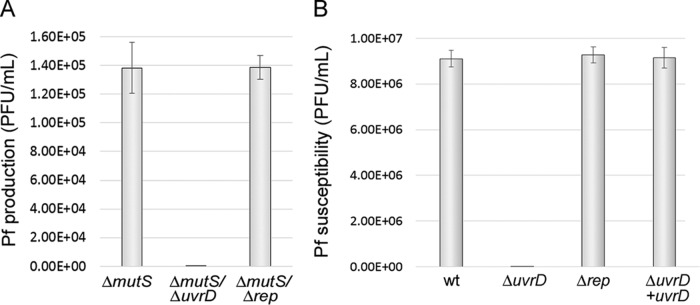
Pf particles are unable to replicate in a Δ*uvrD* mutant of *P. aeruginosa*. (A) Spontaneous production of Pf particles was quantified in overnight cultures of the strains shown in the graphic. When the *uvrD* gene was deleted from the Δ*mutS* strain, the resultant double mutant Δ*mutS* Δ*uvrD* strain lost the capacity to produce Pf particles. In contrast, no effect of deletion of the *rep* gene from the Δ*mutS* strain was observed. PAO1 strain was used for titration of the phage. (B) The PAO1 wild type (wt) and its derivative Δ*rep* and Δ*uvrD* mutants were infected with a concentrated suspension of SI Pf (~10^7^ phage particles/ml). While wt PAO1 and the Δ*rep* mutant were highly sensitive to the infection with Pf, forming more than 8 × 10^6^ plaques/ml, the Δ*uvrD* mutant was fully resistant. An intact *uvrD* gene was reinserted into the Δ*uvrD* mutant by allelic replacement, and the resultant strain fully recovered the susceptibility to Pf at the wild-type level. Data represent means and standard deviations of the results of 3 independent experiments.

We recently reported that RCR of two filamentous phages of *V. cholerae*, CTXφ and VGJφ, depends on UvrD helicase (12). In contrast, RCR of TLCφ, a defective phage of *V. cholerae*, as well as *E. coli* filamentous phages Ff and Ike depends on the replicative accessory helicase Rep (19). This finding raised the issue of whether the role of UvrD in phage replication is an exception for *V. cholerae* or whether it is an additional function of UvrD extended among bacteria. Thus, we tested the role of UvrD in the replication of *P. aeruginosa* phage Pf. We observed that deletion of *uvrD* gene in a Δ*mutS* genetic background completely arrested spontaneous SI Pf production. In contrast, deletion of the *rep* gene from the Δ*mutS* mutant had no effect on SI Pf production (Fig. 3A). Additionally, we exposed a single mutant *ΔuvrD* strain to a concentrated suspension of SI Pf isolated from the supernatant of the *ΔmutS* strain (~10^6^ particles/ml). As expected, the *ΔuvrD* mutant was refractory to infection with SI-Pf whereas strain PAO1 and the *Δrep* strain were highly susceptible (Fig. 3B). The reinsertion of a wt *uvrD* copy into the *ΔuvrD* strain reestablished the susceptibility to SI Pf infection (Fig. 3B). These results demonstrated that UvrD plays an essential role in Pf phage replication.

### The histone-like HU protein contributes to Pf replication.

**FIG 4:**
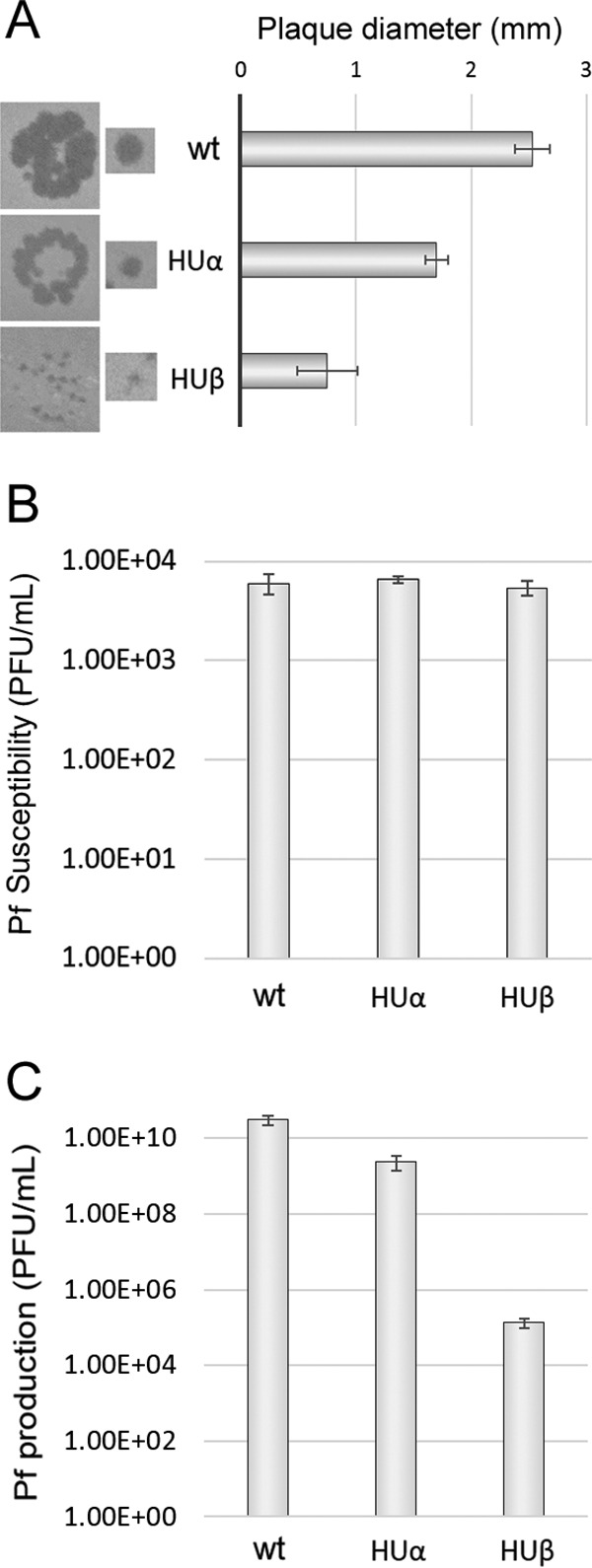
Quantification of contribution of HUα and HUβ to Pf replication. (A) Plaques produced after infection of strains HUα and HUβ with Pf are smaller than plaques formed by wt PAO1. The left panels show pictures of representative plaques of each strain, and the graphic to the right shows the average diameter of plaques for each strain. (B) Graphic of the susceptibility of strains HUα and HUβ after they were exposed to SI Pf particles at a multiplicity of infection (MOI) of 10^−4^ phages/cell. Both strains showed levels of susceptibility to Pf similar to those seen with the wt PAO1 strain. (C) Pf-infected PAO1, HUα, and HUβ cells were allowed to produce Pf particles overnight, and the amounts of particles produced in the medium were measured by titration. As shown in the graph, the level of particles produced by the HUβ strain was 5 orders of magnitude lower than that seen with the PAO1 strain, while the level of particles produced by the HUα strain was 1 order of magnitude lower. Data represent means and standard deviations of the results of 3 independent experiments.

We previously found that, in addition to UvrD, HU is a host factor involved on CTXφ and VGJφ replication in *V. cholerae* (12). In order to test whether the similarity between Pf and *V. cholerae* phages is not limited to UvrD-mediated replication but also includes the use of other host factors in the replication process, we tested the role of HU in Pf replication. HUα and HUβ transposon insertion mutants from the University of Washington mutant collection (20) were used for this purpose. SI Pf was able to generate plaques on lawns of both HUα-deficient and HUβ-deficient strains. However, the plaques observed on lawns of the HUβ strain were visibly smaller than those formed on lawns of HUα, which at the same time were smaller than the plaques formed by the parental strain (0.75 versus 1.70 versus 2.50 mm in diameter, respectively) (Fig. 4A). This observation suggests that replication of Pf, as in the case of CTXφ, is partially affected by the lack of HUβ and to a lesser extent by the lack of HUα.

To quantitate the impact of HU on Pf replication, we measured the capacity of HUα-deficient and HUβ-deficient strains to produce Pf in overnight lysogeny broth (LB) cultures. We first discarded the idea of a possible role of HU in Pf internalization that could affect the phage propagation. HUα and HUβ exposed to SI Pf at a low multiplicity of infection (~10^−4^) showed susceptibility to the phage equal to that seen with the wt PAO1 strain (Fig. 4B). Then, we measured the ability of the infected HUα, HUβ, and PAO1 strains to produce SI Pf particles during overnight cultures. HUβ produced around 5 orders of magnitude less SI Pf than the wt and 3 orders less than HUα (Fig. 4C). These results indicate that, as seen with CTXφ, HU plays an important role in Pf replication. Also, similarly to the case of CTXφ, the contribution of HUβ is more important than that of HUα.

### The PA0727 ORF encodes the Pf initiator protein.

**FIG 5:**
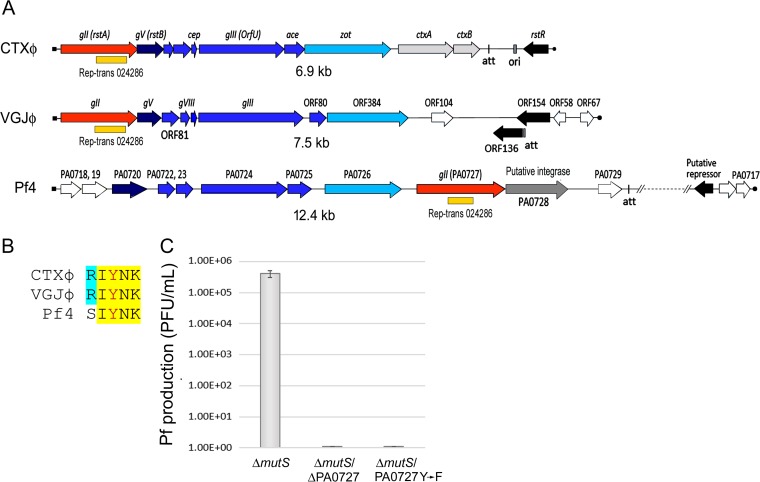
Identification of the gene encoding the rolling circle initiator protein of Pf. (A) Genomic organization of Pf phage compared with that of CTXφ and VGJφ phages. The linear maps of CTXφ, VGJφ, and Pf phages were aligned using the structural (deep blue) and assembly (cerulean blue) gene modules, which conserve the same genome organization in the three phages. The replication initiator protein (IP) genes (in orange) conserved their relative positions in CTXφ and VGJφ phages but not in Pf, where its relative positions shifted. A conserved domain of the family Rep-trans 024286 inside the IP genes is shown with a yellow bar. Other notable genes, such as those encoding repressors (in black), single-stranded DNA (ssDNA) binding proteins (dark blue), cholera toxin in CTXφ phage (pale gray), and Pf integrase (dark gray), are shown. Other genes of unknown function are represented in white. The site of site-specific recombination with the bacterial chromosome (*attP*) is also shown. A fragment of the Pf genome (represented by a dashed segment) was omitted to simplify the scheme. (B) Alignment of the catalytic sites of IPs from CTXφ, VGJφ, and Pf phages. Identical amino acids in the three phages are highlighted in yellow, where the catalytic tyrosine residue is shown in red. (C) Graph showing the suppression of Pf phage production after deletion of the PA0727 ORF from the Δ*mutS* strain and after changing of the catalytic tyrosine residue of PA0727 to a phenylalanine in the Δ*mutS* strain (PA0727Y → F). Data represent means and standard deviations of the results of 3 independent experiments.

A comparative analysis of the DNA sequences of the Pf genome did not reveal any obvious ORF encoding an initiator protein with homology to those of other filamentous phages. In addition, Pf does not have a genome organization similar to those of other filamentous phages which may help to identify the IP-encoding gene by its conserved position inside the phage genome (Fig. 5A). In the region where we expected to find the gene encoding the IP, Pf contains several small ORFs with unidentified functions (Fig. 5A). However, analysis of the protein encoded by PA0727, an ORF localized upstream of the putative Pf integrase, revealed that it contains a Pfam02486 domain and a catalytic module, SIYNK, which is also present in the IPs of CTXφ and VGJφ (Fig. 5B). To test a possible role of the PA0727 product in Pf replication, we deleted the PA0727 gene (Table 1) of the Pf-integrated copy in a Δ*mutS* background. Deletion of the PA0727 gene completely abolished production of SI Pf in the Δ*mutS* mutant (Fig. 5C). To discard any collateral effect of the PA0727 deletion on the Pf life cycle besides replication, we introduced a direct mutation replacing the putative catalytic tyrosine at position 223 of the PA0227 product (PA2077 Y → F). Consistent with a role of the PA0727 product in Pf replication, the 223 Y → F substitution completely abolished production of SI Pf particles in the Δ*mutS* background (Fig. 5C). These results, taken together, indicate that PA0727 is the IP of Pf and that the tyrosine at position 223 catalyzes the initial nick at the origin of replication during RCR.

### Dependence on UvrD for filamentous phage replication may be extensive among bacteria.

As mentioned above, the IPs of CTXφ, VGJφ, and Pf, which rely on UvrD and HU for replication, have structural similarities in the same way that the IPs of TLCφ, Ff, and Ike, which replicate independently of HU and depend on the Rep helicase activity, also share structural similarities. This observation revealed a correlation between the phage IP structure and host factors mediating phage replication. Taking this into account, we made an extended analysis of the IPs from most of the filamentous phages described so far to predict potential host factors involved in filamentous phage replication in other bacteria. We found that a Pfam02486-like domain which is conserved in the IP structure of CTXφ, VGJφ, and Pf is also present in the IPs of several filamentous phages of diverse bacterial species (Table 2). However, IPs containing a Pfam05144 or Pfam05155 domain seem to be confined to the prototypical filamentous phages of *E. coli* and TLCφ of *V. cholerae*. This observation suggests that the role of UvrD in filamentous phage replication is more extensive in bacteria than we had previously thought. The analysis also revealed the existence of a third group of filamentous phages containing a Pfam1446-like domain. Those proteins harbor a highly conserved catalytic module, VGYVAKY (Table 2). Interestingly, the Pfam1446 domain is also found in the IP structure of the φX174 lytic phage of *E. coli*. This phage replicates by a similar RCR mechanism using the Rep helicase. This suggests that this third group of filamentous phages may depend on Rep helicase too.

**TABLE 2:** Initiator proteins of filamentous phages^a^

Phage(s)	Host	Predicted IP domain(s)	Catalytic module^b^	Reference(s)
CTXφ	*V. cholerae*	Pfam02486	SRI**Y**WRI**Y**NKA	36
VGJφ/VEJφ	*V. cholerae*	Pfam02486	SAI**Y**WRI**Y**NKk	37, 38
VSK	*V. cholerae*	Pfam02486	SAI**Y**WRI**Y**NKK	39
KSF-1	*V. cholerae*	Pfam02486	SAI**Y**WRI**Y**NKK	40
FS1	*V. cholerae*	Pfam02486	SAI**Y**WRI**Y**NKK	41
VCYφ	*V. cholerae*	Pfam02486	Undetermined	42
Vf12/Vf33	*V. parahaemolyticus*	Pfam02486	SRI**Y**WRI**Y**NKA	43
RS1/RSS1	*R. solanacearum*	Pfam02486	Undetermined	44
Pf	*P. aeruginosa*	Pfam02486	NGLQLSI**Y**NKT	45
TLCφ	*V. cholerae*	Pfam05144 and Pfam05145	LCI**Y**TKH	46
f1/M13/Fd	*E. coli*	Pfam05144 and Pfam05145	LVA**Y**lKH	47–49
Ike	*E. coli*	Pfam05144 and Pfam05145	LVA**Y**lKH	50
IF1	*E. coli*	Pfam05144 and Pfam05145	Undetermined	51
FS2	*V. cholerae*	Pfam05840	AVG**Y**VAK**Y**LSK	52
VFJφ	*V. cholerae*	Pfam1446	AVG**Y**VAK**Y**LSK	53
φLF	*X. campestris*	Pfam1446	CVG**Y**LAK**Y**ASK	54
Cf1c/Cf16	*X. campestris*	Pfam1446	CVG**Y**LAK**Y**ASK	55

a This is not a complete list of the described filamentous phages but includes most of those with identified IPs. *X. campestris*, *Xanthomonas campestris.*

b Undetermined, the IP sequence did not reveal a conserved catalytic module. Predicted catalytic tyrosines are underlined and highlighted in bold.

## DISCUSSION

Prototypical filamentous phages of *E. coli* use the replicative accessory helicase Rep for rolling circle replication (19). However, here we report for the first time that replication of Pf filamentous phage of *P. aeruginosa* depends on the DNA repair UvrD helicase. Rep and UvrD are two members of the SF1 family of helicases and share approximately 40% amino acid sequence similarity (21). However, despite their similarities, the physiological roles of UvrD and Rep are not interchangeable (22, 23). Rep directly interacts with the bacterial DnaB helicase in *E. coli* and provides a second motor which helps the replisome progress along highly transcribed regions of the chromosome (13, 14). Rep is also involved in the restart of stalled replication forks after replication has been interrupted (24). Consistently, deletion of Rep in *E. coli*, as well as in *V. cholerae*, has been shown to significantly reduce the rate of chromosome replication and consequently the growth rate (22, 23). On the other hand, UvrD plays a pivotal role in MutHLS-dependent mismatch DNA repair (MMR) (16) and UvrABC-dependent nucleotide excision repair (NER) in *E. coli* (15). The role of UvrD in NER in *P. aeruginosa* has not been determined. Oliver et al. reported that a strain harboring an UvrD variant with a substitution of 3 amino acids in the ATP binding site showed a deficient MMR phenotype but UV sensitivity equal to that of the wild type (18). In contrast, here we show that deletion of the PA5443 gene leads to a UV-sensitive phenotype, indicating that, in *P. aeruginosa*, UvrD is also involved in the UvrABC-dependent nucleotide excision repair pathway in a manner similar to that seen with *E. coli* (Fig. 1A and B). This result suggests that the UvrD variant isolated by Oliver et al. does not completely arrest the ATP hydrolysis activity of UvrD but that it is affected in a way that mainly impairs the MMR function and retains the NER activity. Thus, the active role of *P. aeruginosa* UvrD in both MMR and NER reveals for the first time that the PA5443 gene encodes a fully functional homologue of *E. coli* UvrD in *P. aeruginosa.*

In *E. coli*, UvrD also promotes the movement of the replisome along regions of protein-DNA complexes and participates in the restart of replication forks after a stall caused by encountering protein-bound DNA (14). However, the role of UvrD in chromosome replication seems to be more limited than the role of the replicative Rep helicase. While a Rep deletion mutant seriously impairs growth, a deletion mutant of *uvrD* exhibited a normal growth phenotype (13). Nevertheless, a previous study demonstrated that the UvrD activity, as in the case of Rep, can promote progression of replisomes. Some heterologous RCR plasmids, most of them isolated from Gram-positive bacteria, can replicate in *E. coli* using UvrD (25). Replication of these plasmids in the original bacteria depends on the unique accessory helicase PcrA (26). More recently, we described a role for UvrD in RCR under natural conditions. UvrD assists replication of the filamentous phages CTXφ and VGJφ in *V. cholerae* (12).

The implication of Rep helicase in RCR of filamentous phages was reported in the early 1970s, when the molecular biology of the Ff family of phages was extensively studied in *E. coli* (19). This function of Rep is consistent with its role on chromosome replication. More recently, many other filamentous phages were identified in several bacteria, aided by the advent of the deep sequencing technologies. Association of Rep helicase with replication of filamentous phages was probably assumed from its role in replication of filamentous phage of *E. coli*. However, the findings on the role of UvrD in CTXφ and VGJφ replication revealed that the function of Rep in filamentous phage replication was not a paradigm. This finding raised the issue of whether the role of UvrD in RCR of filamentous phages was restricted to *V. cholerae* bacteria or whether it is a more generalized function of UvrD among bacteria. Thus, we examined the capacities of UvrD versus Rep to support rolling circle replication of the clinically relevant *P. aeruginosa* filamentous phage Pf.

*P. aeruginosa* is evolutionarily distant from *V. cholerae* and *E. coli*. Accordingly, filamentous phages of *P. aeruginosa* show remarkable differences from those of *V. cholerae* and *E. coli*. The evolutionary distance of Pf is especially notable by the lack of an orthodox genomic modular organization, which is conserved among most of the filamentous phages described to date (Fig. 1A). In terms of the biological cycle, Pf is also remarkably different from other filamentous phages. While most of them exploit XerCD recombinases for integration in the *dif* site of the bacterial chromosome (9), Pf integrates in the tRNA-*gly* gene, probably using its own encoded integrase. Additionally, under certain conditions Pf is induced and forms a superinfective variant of the phage (SI Pf), which is able to infect *P. aeruginosa* even when it contains a copy of Pf integrated into the chromosome. This process is called superinfection because it circumvents the phage immunity normally provided by a resident lysogenized phage (27). Even more interesting, the SI-Pf variant, unlike previous described filamentous phages, can kill the host bacteria by an unknown mechanism (5).

From this study, we report that Pf is continually produced in the supernatant of a Δ*mutS* strain. The mechanism by which Pf is induced in the Δ*mutS* strain remains unclear. However, the fact that deletion of *mutS* completely arrests the MMR system and leads to a hypermutagenic phenotype suggests that some mutation in the genome of the phage derepresses the excision and replicative functions of Pf. This idea is supported by a study that revealed a high frequency of mutation in the Pf genome in biofilms, where SI Pf is normally produced (28). We found that deletion of *uvrD* gene completely abolished production of SI Pf variants in a Δ*mutS* genetic background. This finding suggested that Pf replication depends on the activity of the UvrD helicase. Accordingly, deletion of *uvrD* rendered a strain fully resistant to SI-Pf (Fig. 3). Complementation of the Δ*uvrD* strain with a wt copy of *uvrD* reestablished the susceptibility to SI Pf infection (Fig. 3). Altogether, these results revealed that UvrD is essential for Pf replication.

Here we also report that the histone-like HU protein promotes Pf replication. HU is a major component of the bacterial nucleoid (29). It is a small protein composed of two closely related subunits, HUα and HUβ. HU binds duplex DNA with low affinity and without specificity but recognizes defined DNA structures and repair intermediates with high affinity (30, 31). Although the main form of HU in bacteria is the heterodimer HUαβ, both homodimers (HU2α and HU2β) have been detected (32). We previously developed a screening method which revealed that *V. cholerae* HU is involved in CTXφ phage replication (12). Here we show that HU from *P. aeruginosa* participates in Pf replication. Interestingly, the role of HUβ was more relevant than that of HUα in both cases, suggesting a common mechanism by which HU promotes rolling circle replication in both phages. A previous report noted that HU specifically binds to the origin of an RCR plasmid, pKYM, and enhances binding of the initiator protein (33). A similar role of HU in RCR of filamentous phages is highly probable. Interestingly, *E. coli* filamentous phages and *V. cholerae* TLCφ, which use Rep helicase for replication, replicate independently of HU. Thus, it is tempting to speculate that these two groups of phages harbor two well-defined families of IPs which differ not only in the nature of the interaction with other copartners, as the accessory helicases, but also in the way that they recognize the replication origin in the replicative form of the phage.

**FIG 6:**
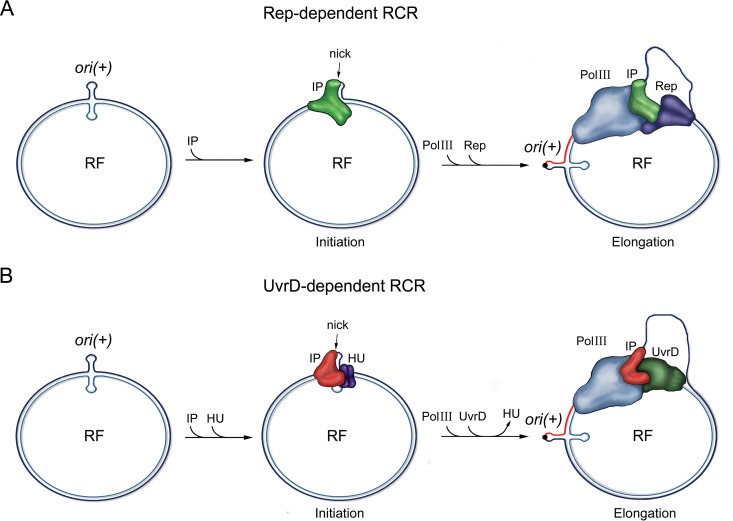
Proposed models for rolling circle replication dependent on Rep or UvrD proteins. (A) The IP of Rep-dependent filamentous phages recognizes the origin of replication, *ori*(*+*), independently of HU and recruits DNA polymerase III (Pol III) and Rep helicase to form the replisome. The IP introduces a nick at the *ori*(*+*) position in the positive DNA strand, which remains attached to the IP by the 5′ end. The replisome starts DNA polymerization by extending the 3′ end of the nick, while Rep helicase opens DNA strands. (B) The IP of phages depending on UvrD requires HU to recognize *ori*(*+*). Subsequently, the IP recruits polymerase III (Pol III) and UvrD helicase in this case to form the replisome. Replication starts and continues as described for panel A. In both cases, the positive DNA strand is represented in dark blue, the negative strand in cerulean blue, and the *de novo* synthesized DNA in red. RF, replicative form.

Additionally, we identified the initiator protein of Pf; the protein is encoded by the PA0727 gene of the PAO1 strain. It contains a predicted Pfam02486 domain and a catalytic module, SIYNK, conserved in the IPs of CTXφ and VGJφ. We tested the function of the tyrosine contained in the SIYNK module by replacing it with a phenylalanine and demonstrated that it is essential for the establishment and production of Pf phage. Interestingly, our analysis of the predicted domain structures of most of the IPs described so far revealed that the Pfam08426 domain is widely distributed among filamentous phages of different bacterial species. In contrast, the Pfam05144 and Pfam05145 domains, conserved in the IPs of filamentous phages that depend on Rep for replication, seem to be limited to the prototypical filamentous phages of *E. coli* and the TLCφ satellite phage of *V. cholerae*. Taken together, our results allow the classification of filamentous phages into at least two groups on the basis of the identity of helicase used for replication: those using UvrD and those using Rep (Fig. 6).

In conclusion, we demonstrated for the first time that UvrD of *P. aeruginosa*, which possesses fully functional MMR and NER activities, also assists rolling circle replication of Pf filamentous phage. Additionally, we provide evidence indicating that UvrD protein plays a general role in assisting rolling circle replication of filamentous phages hosted by such diverse bacterial species as *V. cholerae*, *V. parahaemolyticus*, *P. aeruginosa*, and *Ralstonia solanacearum*. This expanded role of UvrD, previously known as a nonreplicative helicase, concerns not only scientists interested in the molecular biology of inoviruses but also those involved in the study of helicases and their role in DNA replication and repair.

## MATERIALS AND METHODS

### Strains and culture conditions.

Strains, plasmids, and oligonucleotides used in this study are described in Table 1, Table 3, and Table 4, respectively. All strains were grown in LB medium, to which agar was added when solid medium was required. LB agar without NaCl plus 15% sucrose was used to segregate suicide plasmid from merodiploids during construction of mutants by allelic exchange. Antibiotics were added, when necessary, at the following concentrations: ampicillin (Amp), 100 µg/ml; carbenicillin (Cb), 300 µg/ml; spectinomycin (Sp), 100 µg/ml; chloramphenicol (Cm), 34 µg/ml for *E. coli* and 200 µg/ml for *P. aeruginosa*; kanamycin (Kn), 50 µg/ml; and rifampin (Rif), 300 µg/ml.

**TABLE 3:** Relevant plasmids used in this study

Plasmid	Genotype	Reference or source
pUC19	Cloning vector Ap^r*a*^	56
pEX100Tlink	*E. coli*-*P. aeruginosa* shuttle suicide vector	34
pMG002	pEX100T::*mutS*	This study
pMG003	pEX100T::*uvrD*	This study
pMG004	pEX100T::PA0727	This study
pMG005	pEX100T::ΔPA0727	This study
pMG006	pEX100T::PA0727 Y → F	This study
pMG007	pEX100T::Δ*mutS*	This study
pMG008	pEX100T::*rep*	This study
pMG009	pEX100T::Δ*rep*	This study

a Ap^r^, ampicillin resistance.

**TABLE 4:** Oligonucleotides used in this study

Oligonucleotide	Use	Sequence (5′–3′)
EM34	Deletion of *mutS*	TACATCTAGAGAGGATCACCGTGTCATCGA
EM35	Deletion of *mutS*	TACATCTAGAAACACACCTGCCTGCACATC
EM36	Cloning of *mutS* region	TACACCATGGTGCTGCCGGGGATTGAGTCG
EM37	Cloning of *mutS* region	CATAAAGCTTGATGACGAGGGACTGAAGAG
EM38	Replacement of Y → F in PA0727	TAACAAGACCCTCCAGGCTC
EM39	Replacement of Y → F in PA0727	AAGATCGACAGTTGCAGGC
EM40	Deletion of PA0727	GAGTTCTGGCACGTTATCG
EM41	Deletion of PA0727	GTCCGACTCGATACTGACG
EM42	Deletion of *rep*	TACACCATGGACGCCCCGCAAGAGGTCAAG
EM43	Deletion of *rep*	TACACCATGGTGCTGCCGGGGATTGAGTCG
EM44	Cloning of *rep* region	TACAAAGCTTCGGATAGAACTGCGCCCAGG
EM45	Cloning of *rep* region	TACATCTAGATGCAACCCTGGCGGCTCTTG
EM46	Deletion of *uvrD* helicase	TACATCTAGATGGCTGATGCTCGGCTACGC
EM47	Deletion of *uvrD* helicase	TACATCTAGAGCGGGTCGTTGAGGGAGTTC
EM48	Cloning of *uvrD* region	TACAGGATCCCCAGGTCCCAGTCCCACAAC
EM49	Cloning of *uvrD* region	TACAAAGCTTTGTGGCCCTGCTCGGTGATC
EM56	Deletion of PA0727	CATAGGTACCTCATCGAGTACAAGCGGATC
EM57	Deletion of PA0727	TACATCTAGAATCGAACAGCCTCAGATAGG

### DNA isolation and manipulation.

*P. aeruginosa* total DNA was prepared using a GenElute bacterial genomic DNA kit (Sigma). Plasmid DNA was prepared using a QIAprep Spin Miniprep kit (Qiagen), which was used also to purify the replicative form of Pf phage. DNA fragments were purified from agarose gel using a QIAquick gel extraction kit (Qiagen). Restriction and modification enzymes were used according to the manufacturer’s instructions (NE Biolabs). The DNA was electrophoresed on 0.8% (wt/vol) agarose gels and was visualized with ethidium bromide (1 µg/ml).

### Pf identification.

The genome of Pf was sequenced for confirmatory purposes when needed. The replicative form of Pf was digested with restriction enzymes BamHI and HindIII, and the generated DNA fragments were inserted into the pUC19 vector. The resultant constructs were sequenced using universal M13 Fw and Rev primers.

### Construction of *P. aeruginosa* mutants.

All mutant strains were constructed by allelic exchange using suicide vector pEX100Tlink (34). The mutants were confirmed by PCR and sequencing. Complementation of the mutants was performed by replacing the mutated allele with the original copy from the parental strain PAO1, also through allelic exchange. *E. coli* DH5α was the host for plasmid constructions, and *E. coli* S17-1 λpir was used as the donor for conjugative transfer of the suicide vector pEX100Tlink-based constructs into *P. aeruginosa*.

### Isolation of a superinfective variant of Pf.

A MutS-deficient strain which spontaneously produces superinfective Pf (SI Pf) viral particles was used as the SI Pf donor. The strain was grown in 5 ml of LB for 20 h with shaking (240 rpm) at 37°C. One milliliter of the culture was spun down, and the supernatant was serially filtered through 0.45- and 0.22-µm-pore-size filters (sartorius). Cell-free supernatants containing Pf particles were stored at 4°C until use. When concentrated phage preparations were required, the phage particles were precipitated from the filtered supernatant by adding sodium chloride and polyethylene glycol to give final concentrations of 3% and 5% (wt/vol), respectively. The mixture was incubated on ice for 30 min and centrifuged at 12,000 × *g* for 20 min. The supernatant was discarded, and the phage-containing pellet was resuspended in a desired volume of phosphate-buffered saline (PBS). The concentrated suspension was stored at 4°C until use. Identity of the phage was confirmed by sequencing as described above.

### Phage titration.

Titers of SI Pf were routinely determined by dropping serial dilutions of filtered culture supernatants of phage-producing cells onto soft LB agar (0.4%) plates containing the PAO1 strain. The numbers of plaques were determined after overnight incubation at 37°C. Isolated plaques were used to isolate monoclonal SI Pf when required.

### Infection assay.

A 10-μl volume of the cell-free supernatants containing Pf particles was mixed with 20 µl of recipient strains cultured in LB to an optical density (OD) of 0.5. Mixtures were incubated 20 min at room temperature to allow infection and then plated on LB. Infection events were determined by counting the plaques formed on soft LB agar over PAO1 lawns.

### Phage production quantitation.

To quantitate the capacity of strains to produce Pf virions, infected cells were spun down and the supernatant was discarded to avoid counting unabsorbed virions already present in the suspension used for infecting the cells. Cells were suspended in 5 ml of LB and incubated overnight at 37°C. The titers of Pf were determined as described above and normalized against the OD of the cultures tested.

### UV survival assay.

Tested strains were cultured overnight in LB media. One milliliter of the culture was pelleted, cells were suspended in PBS, and the OD of the suspensions was adjusted to 0.5. Bacterial suspensions were exposed to UV irradiation for 0, 2, 4, and 6 s using a UV transilluminator (λ = 305 nm). Serial dilutions of irradiated bacterial suspensions were plated on LB agar to count the amount of surviving cells. Colonies were counted after overnight incubation at 37°C.

### Mutation frequency determination assay.

To determine the mutation frequency of the strains, serial dilutions of overnight cultures were plated onto LB agar in the presence or absence of rifampin (300 µg/ml). Spontaneous appearance of bacteria resistant to rifampin was determined by counting colonies after 36 h of incubation at 37°C. All experiments were performed in triplicate and the mean value and standard deviation calculated.
